# (*S*)-4-Phenyl-2-(1,2,3,4-tetra­hydro­isoquinolin-3-yl)-1,3-thia­zole

**DOI:** 10.1107/S1600536811052585

**Published:** 2011-12-21

**Authors:** Sunayna Pawar, Pralav Bhatt, Thavendran Govender, Hendrik G. Kruger, Glenn E. M. Maguire

**Affiliations:** a School of Pharmacy and Pharmacology, University of KwaZulu-Natal, Durban 4000, South Africa; b School of Chemistry, University of KwaZulu-Natal, Durban 4000, South Africa

## Abstract

In the title compound, C_18_H_16_N_2_S, the N-containing ring adopts a half-chair configuration. The crystal packing features C—H⋯N contacts. There is no π–π stacking within the crystal structure.

## Related literature

The title compound is a potential ligand for the asymmetric Henry reaction. For the application of these ligands as catalysts, see: Chakka *et al.* (2010 ▶); Kawthekar *et al.* (2010 ▶); Peters *et al.* (2010 ▶); Naicker *et al.* (2010 ▶). For related structures, see: Naicker *et al.* (2011*a* ▶,*b* ▶).
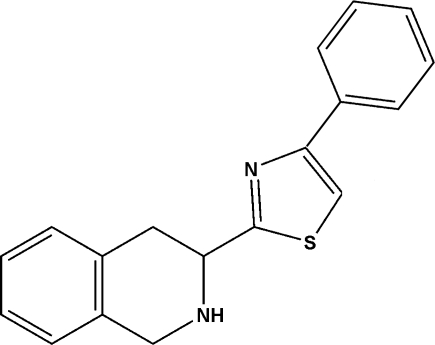


## Experimental

### 

#### Crystal data

C_18_H_16_N_2_S
*M* *_r_* = 292.39
Trigonal, 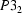

*a* = 16.223 (1) Å
*c* = 4.8130 (3) Å
*V* = 1097.0 (1) Å^3^
*Z* = 3
Mo *K*α radiation
μ = 0.22 mm^−1^
*T* = 173 K
0.20 × 0.10 × 0.09 mm

#### Data collection

Bruker Kappa DUO APEXII diffractometer
Absorption correction: multi-scan (*SADABS*; Sheldrick, 2008 ▶) *T*_min_ = 0.958, *T*_max_ = 0.981
14735 measured reflections
3676 independent reflections
3205 reflections with *I* > 2σ(*I*)
*R*_int_ = 0.030

#### Refinement

*R*[*F* ^2^ > 2σ(*F* ^2^)] = 0.035
*wR*(*F* ^2^) = 0.084
*S* = 1.03
3676 reflections
194 parameters
1 restraint
H atoms treated by a mixture of independent and constrained refinement
Δρ_max_ = 0.23 e Å^−3^
Δρ_min_ = −0.22 e Å^−3^
Absolute structure: Flack (1983 ▶), 1832 Friedel pairs
Flack parameter: −0.02 (6)

### 

Data collection: *APEX2* (Bruker, 2006 ▶); cell refinement: *SAINT* (Bruker, 2006 ▶); data reduction: *SAINT*; program(s) used to solve structure: *SHELXS97* (Sheldrick, 2008 ▶); program(s) used to refine structure: *SHELXL97* (Sheldrick, 2008 ▶); molecular graphics: *OLEX2* (Dolomanov *et al.*, 2009 ▶); software used to prepare material for publication: *SHELXL97*.

## Supplementary Material

Crystal structure: contains datablock(s) I, global. DOI: 10.1107/S1600536811052585/gw2110sup1.cif

Structure factors: contains datablock(s) I. DOI: 10.1107/S1600536811052585/gw2110Isup2.hkl

Supplementary material file. DOI: 10.1107/S1600536811052585/gw2110Isup3.cml

Additional supplementary materials:  crystallographic information; 3D view; checkCIF report

## Figures and Tables

**Table 1:** Hydrogen-bond geometry (Å, °)

*D*—H⋯*A*	*D*—H	H⋯*A*	*D*⋯*A*	*D*—H⋯*A*
C11—H11⋯N1^i^	0.95	2.54	3.341 (3)	142

Symmetry code: (i) 
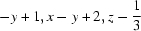
.

**Fig. 1.**
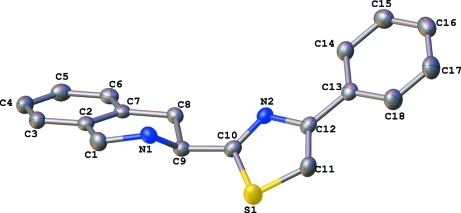
The molecular structure of the title compound with the atom numbering scheme. Displacement ellipsoids are drawn at the 40% probability level. The hydrogen atoms have been omitted for clarity.

**Fig. 2.**
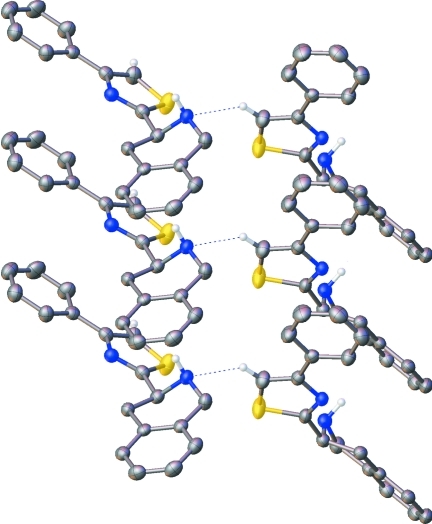
CH···N interactions along the b axis.

## supplementary crystallographic information

### Comment

Tetrahydroisoquinoline is a core structure in various natural and pharmaceutically active compounds, displaying a broad spectrum of activity. Molecules with a C_1_-symmetric tetrahydroisoquinoline backbone have been studied for various catalytic reactions such as allylic alkylation (Blanc *et al.*,2003*)*, Henry reactions (Kawthekar *et al.*, 2010), asymmetric hydrogenation reactions (Chakka *et al.*,2010; Peters *et al.*, 2010) and Diels-Alder reactions (Naicker *et al.*,2010). The title compound is one of the ligands used for asymmetric Henry reaction, its' catalytic activity is currently under investigation. The chiral carbon (C9) has been assigned an *S* configuration from two-dimensional NMR measurements.

In the title compound, the piperidine ring of the tetrahydroisoquinolinone unit adopts a half chair (Fig 1). This hetrocyclic ring within similar structures displays either a half chair or half boat conformation (Naicker *et al.* 2011*a*; 2011*b*). From the crystal structure it is evident that the *N*-containing six membered ring assumes a half chair conformation [Q = 0.483 (2) Å, θ = 50.1 (2)° and φ = 321.8 (3)°]. The torsion angle for C1—N1—C9—C10 is -171.72 (14)°. From the plain formed by the atoms C1—C2—C7—C8—C9—N1 the maximum displacement from planarity for N1 is 0.297 Å and for C9 0.331 Å (Fig. 1). This is similar to our previously reported structures which also assume half chair conformations (Naicker *et al.*, 2011*a*; 2011*b*). The crystal packing contains C—H···N contacts of distance 3.341 (3) Å (see Fig. 2) (Table 1). There is no π-π stacking within the crystal structure.

### Experimental

The *N*-protected thiazole (3 mmol) was dissolved in THF (15 ml), to this 12 *M* HCl (15 ml) was added slowly and the reaction mixture was stirred at room temperature for 2 h. THF was evaporated under vacuum. The reaction was monitored by TLC using EtOAc/Hexane (20:80, *R*_f_ = 0.5). The reaction mixture was slowly poured into aqueous saturated NaHCO_3_ solution, the mixture was then extracted with CH_2_Cl_2_ (3 *x* 30 ml). The combined organic layer was dried over MgSO_4_. The solvent was evaporated under reduced pressure, the residue was purified by column chromatography on silica gel (deactivated with 5% Et_3_N) with Et_3_N/EtOAc/Hexane (5/8/100) as the eluent to afford the thiazole as a yellow solid (0.27 g, yield 95%). *M*.p. = 357–360 K.

Recrystallization from tetrahydrofuran at room temperature afforded colourless crystals suitable for X-ray analysis.

### Refinement

All non-hydrogen atoms were refined anisotropically. All hydrogen atoms, except H1N, were positioned geometrically with C—H distances ranging from 0.95 Å to 1.00 Å and refined as riding on their parent atoms with *U*_iso_ (H) = 1.2 - 1.5 *U*_eq_ (C).

### Crystal data

**Table:**

C_18_H_16_N_2_S	*D*_x_ = 1.328 Mg m^−^^3^
*M**_r_* = 292.39	Mo *K*α radiation, λ = 0.71073 Å
Trigonal, *P*3_2_	Cell parameters from 14735 reflections
Hall symbol: P 32	θ = 2.5–28.4°
*a* = 16.223 (1) Å	µ = 0.22 mm^−^^1^
*c* = 4.8130 (3) Å	*T* = 173 K
*V* = 1097.0 (1) Å^3^	Needle, yellow
*Z* = 3	0.20 × 0.10 × 0.09 mm
*F*(000) = 462	

### Data collection

**Table:**

Bruker Kappa DUO APEXII diffractometer	3676 independent reflections
Radiation source: fine-focus sealed tube	3205 reflections with *I* > 2σ(*I*)
graphite	*R*_int_ = 0.030
0.5° φ scans and ω scans	θ_max_ = 28.4°, θ_min_ = 2.5°
Absorption correction: multi-scan (*SADABS*; Sheldrick, 2008)	*h* = −21→21
*T*_min_ = 0.958, *T*_max_ = 0.981	*k* = −21→21
14735 measured reflections	*l* = −6→6

### Refinement

**Table:**

Refinement on *F*^2^	Secondary atom site location: difference Fourier map
Least-squares matrix: full	Hydrogen site location: inferred from neighbouring sites
*R*[*F*^2^ > 2σ(*F*^2^)] = 0.035	H atoms treated by a mixture of independent and constrained refinement
*wR*(*F*^2^) = 0.084	*w* = 1/[σ^2^(*F*_o_^2^) + (0.0433*P*)^2^ + 0.1159*P*] where *P* = (*F*_o_^2^ + 2*F*_c_^2^)/3
*S* = 1.03	(Δ/σ)_max_ < 0.001
3676 reflections	Δρ_max_ = 0.23 e Å^−^^3^
194 parameters	Δρ_min_ = −0.22 e Å^−^^3^
1 restraint	Absolute structure: Flack (1983), 1832 Friedel pairs
Primary atom site location: structure-invariant direct methods	Flack parameter: −0.02 (6)

### Special details

**Table:**

Experimental. Half sphere of data collected using the Bruker *SAINT* software package. Crystal to detector distance = 30 mm; combination of φ and ω scans of 0.5°, 60 s per °, 2 iterations.
Geometry. All e.s.d.'s (except the e.s.d. in the dihedral angle between two l.s. planes) are estimated using the full covariance matrix. The cell e.s.d.'s are taken into account individually in the estimation of e.s.d.'s in distances, angles and torsion angles; correlations between e.s.d.'s in cell parameters are only used when they are defined by crystal symmetry. An approximate (isotropic) treatment of cell e.s.d.'s is used for estimating e.s.d.'s involving l.s. planes.
Refinement. Refinement of *F*^2^ against ALL reflections. The weighted *R*-factor *wR* and goodness of fit *S* are based on *F*^2^, conventional *R*-factors *R* are based on *F*, with *F* set to zero for negative *F*^2^. The threshold expression of *F*^2^ > σ(*F*^2^) is used only for calculating *R*-factors(gt) *etc*. and is not relevant to the choice of reflections for refinement. *R*-factors based on *F*^2^ are statistically about twice as large as those based on *F*, and *R*- factors based on ALL data will be even larger.

### Fractional atomic coordinates and isotropic or equivalent isotropic displacement parameters (Å^2^)

**Table:**

	*x*	*y*	*z*	*U*_iso_*/*U*_eq_	
S1	0.00297 (4)	0.88075 (3)	0.01223 (14)	0.04813 (15)	
N1	−0.22129 (10)	0.70793 (10)	0.1094 (3)	0.0316 (3)	
H1N	−0.2357 (14)	0.6647 (15)	−0.032 (4)	0.036 (5)*	
N2	−0.01656 (9)	0.71580 (9)	−0.0576 (3)	0.0287 (3)	
C1	−0.30267 (13)	0.67560 (13)	0.2960 (4)	0.0359 (4)	
H1A	−0.3603	0.6569	0.1825	0.043*	
H1B	−0.2926	0.7298	0.4142	0.043*	
C2	−0.32126 (11)	0.59286 (11)	0.4831 (4)	0.0287 (3)	
C3	−0.40599 (13)	0.54595 (12)	0.6353 (4)	0.0359 (4)	
H3	−0.4520	0.5655	0.6174	0.043*	
C4	−0.42359 (13)	0.47165 (13)	0.8110 (4)	0.0393 (4)	
H4	−0.4816	0.4402	0.9122	0.047*	
C5	−0.35671 (14)	0.44283 (12)	0.8399 (4)	0.0385 (4)	
H5	−0.3688	0.3915	0.9599	0.046*	
C6	−0.27209 (13)	0.48947 (12)	0.6927 (4)	0.0323 (4)	
H6	−0.2260	0.4702	0.7145	0.039*	
C7	−0.25347 (12)	0.56434 (11)	0.5129 (3)	0.0272 (3)	
C8	−0.16028 (11)	0.61485 (12)	0.3565 (4)	0.0290 (3)	
H8A	−0.1630	0.5759	0.1945	0.035*	
H8B	−0.1080	0.6222	0.4791	0.035*	
C9	−0.13971 (12)	0.71258 (12)	0.2570 (4)	0.0307 (4)	
H9	−0.1268	0.7538	0.4244	0.037*	
C10	−0.05355 (12)	0.75944 (11)	0.0730 (4)	0.0300 (3)	
C11	0.08088 (13)	0.87065 (13)	−0.2040 (4)	0.0388 (4)	
H11	0.1314	0.9222	−0.3007	0.047*	
C12	0.06065 (11)	0.77863 (11)	−0.2186 (4)	0.0297 (3)	
C13	0.11195 (12)	0.74191 (12)	−0.3839 (4)	0.0304 (4)	
C14	0.08499 (13)	0.64602 (13)	−0.3768 (4)	0.0362 (4)	
H14	0.0336	0.6036	−0.2616	0.043*	
C15	0.13257 (14)	0.61174 (14)	−0.5369 (5)	0.0433 (4)	
H15	0.1135	0.5460	−0.5304	0.052*	
C16	0.20739 (14)	0.67241 (16)	−0.7052 (5)	0.0468 (5)	
H16	0.2398	0.6489	−0.8150	0.056*	
C17	0.23454 (16)	0.76791 (16)	−0.7122 (5)	0.0506 (5)	
H17	0.2860	0.8102	−0.8275	0.061*	
C18	0.18785 (15)	0.80224 (15)	−0.5542 (4)	0.0435 (5)	
H18	0.2076	0.8681	−0.5611	0.052*	

### Atomic displacement parameters (Å^2^)

**Table:**

	*U*^11^	*U*^22^	*U*^33^	*U*^12^	*U*^13^	*U*^23^
S1	0.0455 (3)	0.0274 (2)	0.0703 (3)	0.0174 (2)	0.0198 (3)	0.0061 (2)
N1	0.0336 (7)	0.0316 (7)	0.0329 (8)	0.0188 (6)	0.0027 (6)	−0.0008 (6)
N2	0.0263 (7)	0.0257 (7)	0.0299 (7)	0.0099 (6)	0.0002 (6)	0.0010 (6)
C1	0.0344 (9)	0.0346 (9)	0.0433 (10)	0.0208 (8)	0.0042 (8)	0.0016 (8)
C2	0.0280 (8)	0.0268 (8)	0.0284 (8)	0.0116 (7)	−0.0020 (7)	−0.0058 (7)
C3	0.0294 (8)	0.0337 (9)	0.0423 (10)	0.0141 (7)	0.0030 (8)	−0.0054 (8)
C4	0.0344 (9)	0.0319 (9)	0.0413 (10)	0.0088 (7)	0.0103 (8)	−0.0014 (8)
C5	0.0463 (11)	0.0280 (8)	0.0348 (10)	0.0138 (8)	0.0055 (8)	0.0025 (7)
C6	0.0372 (9)	0.0299 (8)	0.0300 (9)	0.0169 (7)	−0.0014 (7)	−0.0031 (7)
C7	0.0291 (8)	0.0255 (7)	0.0243 (8)	0.0117 (6)	−0.0010 (6)	−0.0058 (6)
C8	0.0303 (8)	0.0295 (8)	0.0290 (8)	0.0163 (7)	0.0008 (7)	−0.0004 (7)
C9	0.0321 (8)	0.0278 (8)	0.0314 (9)	0.0143 (7)	0.0012 (7)	−0.0026 (7)
C10	0.0293 (8)	0.0241 (8)	0.0332 (9)	0.0106 (6)	−0.0004 (7)	0.0027 (7)
C11	0.0307 (9)	0.0323 (9)	0.0492 (11)	0.0127 (7)	0.0090 (8)	0.0062 (8)
C12	0.0246 (7)	0.0282 (8)	0.0324 (9)	0.0102 (6)	−0.0018 (7)	0.0026 (7)
C13	0.0259 (7)	0.0331 (8)	0.0292 (9)	0.0126 (7)	−0.0028 (7)	0.0006 (7)
C14	0.0316 (9)	0.0334 (9)	0.0422 (10)	0.0152 (7)	0.0045 (8)	0.0038 (8)
C15	0.0432 (10)	0.0385 (10)	0.0516 (12)	0.0230 (9)	0.0000 (9)	−0.0032 (9)
C16	0.0408 (10)	0.0552 (12)	0.0475 (12)	0.0263 (10)	0.0019 (9)	−0.0101 (10)
C17	0.0432 (11)	0.0513 (12)	0.0493 (13)	0.0177 (10)	0.0186 (10)	0.0060 (10)
C18	0.0424 (10)	0.0369 (10)	0.0456 (11)	0.0157 (8)	0.0133 (9)	0.0078 (9)

### Geometric parameters (Å, °)

**Table:**

S1—C11	1.707 (2)	C7—C8	1.512 (2)
S1—C10	1.7306 (17)	C8—C9	1.525 (2)
N1—C1	1.460 (2)	C8—H8A	0.9900
N1—C9	1.470 (2)	C8—H8B	0.9900
N1—H1N	0.92 (2)	C9—C10	1.501 (2)
N2—C10	1.297 (2)	C9—H9	1.0000
N2—C12	1.390 (2)	C11—C12	1.361 (2)
C1—C2	1.516 (2)	C11—H11	0.9500
C1—H1A	0.9900	C12—C13	1.475 (2)
C1—H1B	0.9900	C13—C14	1.390 (2)
C2—C7	1.397 (2)	C13—C18	1.393 (3)
C2—C3	1.400 (2)	C14—C15	1.389 (3)
C3—C4	1.380 (3)	C14—H14	0.9500
C3—H3	0.9500	C15—C16	1.380 (3)
C4—C5	1.387 (3)	C15—H15	0.9500
C4—H4	0.9500	C16—C17	1.383 (3)
C5—C6	1.386 (3)	C16—H16	0.9500
C5—H5	0.9500	C17—C18	1.372 (3)
C6—C7	1.396 (2)	C17—H17	0.9500
C6—H6	0.9500	C18—H18	0.9500
			
C11—S1—C10	89.46 (9)	H8A—C8—H8B	108.0
C1—N1—C9	110.51 (13)	N1—C9—C10	109.12 (13)
C1—N1—H1N	110.0 (13)	N1—C9—C8	112.02 (13)
C9—N1—H1N	106.0 (13)	C10—C9—C8	112.09 (13)
C10—N2—C12	111.30 (14)	N1—C9—H9	107.8
N1—C1—C2	115.29 (14)	C10—C9—H9	107.8
N1—C1—H1A	108.5	C8—C9—H9	107.8
C2—C1—H1A	108.5	N2—C10—C9	125.27 (15)
N1—C1—H1B	108.5	N2—C10—S1	114.34 (13)
C2—C1—H1B	108.5	C9—C10—S1	120.36 (12)
H1A—C1—H1B	107.5	C12—C11—S1	110.66 (14)
C7—C2—C3	119.19 (16)	C12—C11—H11	124.7
C7—C2—C1	120.86 (14)	S1—C11—H11	124.7
C3—C2—C1	119.92 (15)	C11—C12—N2	114.22 (16)
C4—C3—C2	120.90 (17)	C11—C12—C13	126.56 (16)
C4—C3—H3	119.5	N2—C12—C13	119.21 (14)
C2—C3—H3	119.5	C14—C13—C18	118.28 (17)
C3—C4—C5	120.06 (17)	C14—C13—C12	120.88 (16)
C3—C4—H4	120.0	C18—C13—C12	120.83 (16)
C5—C4—H4	120.0	C15—C14—C13	120.51 (17)
C6—C5—C4	119.54 (17)	C15—C14—H14	119.7
C6—C5—H5	120.2	C13—C14—H14	119.7
C4—C5—H5	120.2	C16—C15—C14	120.46 (19)
C5—C6—C7	121.10 (17)	C16—C15—H15	119.8
C5—C6—H6	119.5	C14—C15—H15	119.8
C7—C6—H6	119.5	C15—C16—C17	119.16 (19)
C6—C7—C2	119.21 (15)	C15—C16—H16	120.4
C6—C7—C8	120.20 (15)	C17—C16—H16	120.4
C2—C7—C8	120.58 (15)	C18—C17—C16	120.65 (19)
C7—C8—C9	111.08 (13)	C18—C17—H17	119.7
C7—C8—H8A	109.4	C16—C17—H17	119.7
C9—C8—H8A	109.4	C17—C18—C13	120.93 (19)
C7—C8—H8B	109.4	C17—C18—H18	119.5
C9—C8—H8B	109.4	C13—C18—H18	119.5
			
C9—N1—C1—C2	−43.4 (2)	C8—C9—C10—N2	19.7 (2)
N1—C1—C2—C7	12.8 (2)	N1—C9—C10—S1	73.07 (17)
N1—C1—C2—C3	−169.22 (16)	C8—C9—C10—S1	−162.25 (13)
C7—C2—C3—C4	−0.7 (3)	C11—S1—C10—N2	0.18 (15)
C1—C2—C3—C4	−178.73 (16)	C11—S1—C10—C9	−178.05 (15)
C2—C3—C4—C5	0.3 (3)	C10—S1—C11—C12	0.02 (16)
C3—C4—C5—C6	0.4 (3)	S1—C11—C12—N2	−0.2 (2)
C4—C5—C6—C7	−0.8 (3)	S1—C11—C12—C13	179.92 (14)
C5—C6—C7—C2	0.4 (3)	C10—N2—C12—C11	0.3 (2)
C5—C6—C7—C8	179.40 (16)	C10—N2—C12—C13	−179.78 (15)
C3—C2—C7—C6	0.3 (2)	C11—C12—C13—C14	178.82 (19)
C1—C2—C7—C6	178.32 (16)	N2—C12—C13—C14	−1.0 (2)
C3—C2—C7—C8	−178.67 (15)	C11—C12—C13—C18	−2.1 (3)
C1—C2—C7—C8	−0.6 (2)	N2—C12—C13—C18	178.05 (16)
C6—C7—C8—C9	−160.07 (15)	C18—C13—C14—C15	−0.3 (3)
C2—C7—C8—C9	18.9 (2)	C12—C13—C14—C15	178.84 (17)
C1—N1—C9—C10	−171.71 (14)	C13—C14—C15—C16	0.0 (3)
C1—N1—C9—C8	63.57 (18)	C14—C15—C16—C17	0.1 (3)
C7—C8—C9—N1	−50.29 (18)	C15—C16—C17—C18	0.0 (3)
C7—C8—C9—C10	−173.34 (13)	C16—C17—C18—C13	−0.2 (3)
C12—N2—C10—C9	177.80 (16)	C14—C13—C18—C17	0.4 (3)
C12—N2—C10—S1	−0.32 (18)	C12—C13—C18—C17	−178.8 (2)
N1—C9—C10—N2	−104.95 (19)		

### Hydrogen-bond geometry (Å, °)

**Table:**

*D*—H···*A*	*D*—H	H···*A*	*D*···*A*	*D*—H···*A*
C11—H11···N1^i^	0.95	2.54	3.341 (3)	142

Symmetry codes: (i) −*y*+1, *x*−*y*+2, *z*−1/3.
